# Supplementary material for: SERINC5 co-expressed with HIV-1 Env or present in a target membrane destabilizes small fusion pores leading to their collapse
Source: mBio. 2025 Nov 17;16(12):e02793-25. doi: 10.1128/mbio.02793-25 (PMC12697135; doi:10.1128/mbio.02793-25)
Supplement: Supplemental Figures — Figures S1–S11. [file mbio.02793-25-s0001.pdf]

Supplementary Figures and Legends

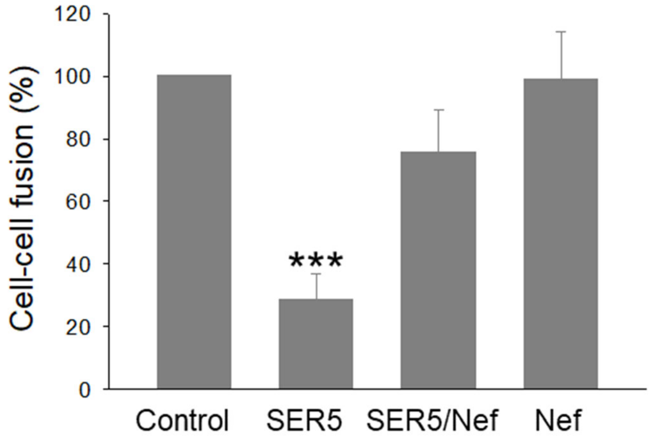

Suppl. Fig. S1. SER5 expressed in effector cells inhibits HIV-1 cell-cell fusion mediated by ADA Env. HeLa cells stably expressing HIV-1 ADA Env were co-incubated with the TZM-bl cells for 3 h at 37 ºC, and the resulting cell-cell fusion quantified by a microscopy-based assay (see Methods). The effector HeLa/ADA cells were transfected with SER5 or NL4.3 Nef alone or with SER5 and Nef, as indicated. Plotted are means and standard deviations from 5 independent experiments. ***, $p<0.001$ (Student's t-test).

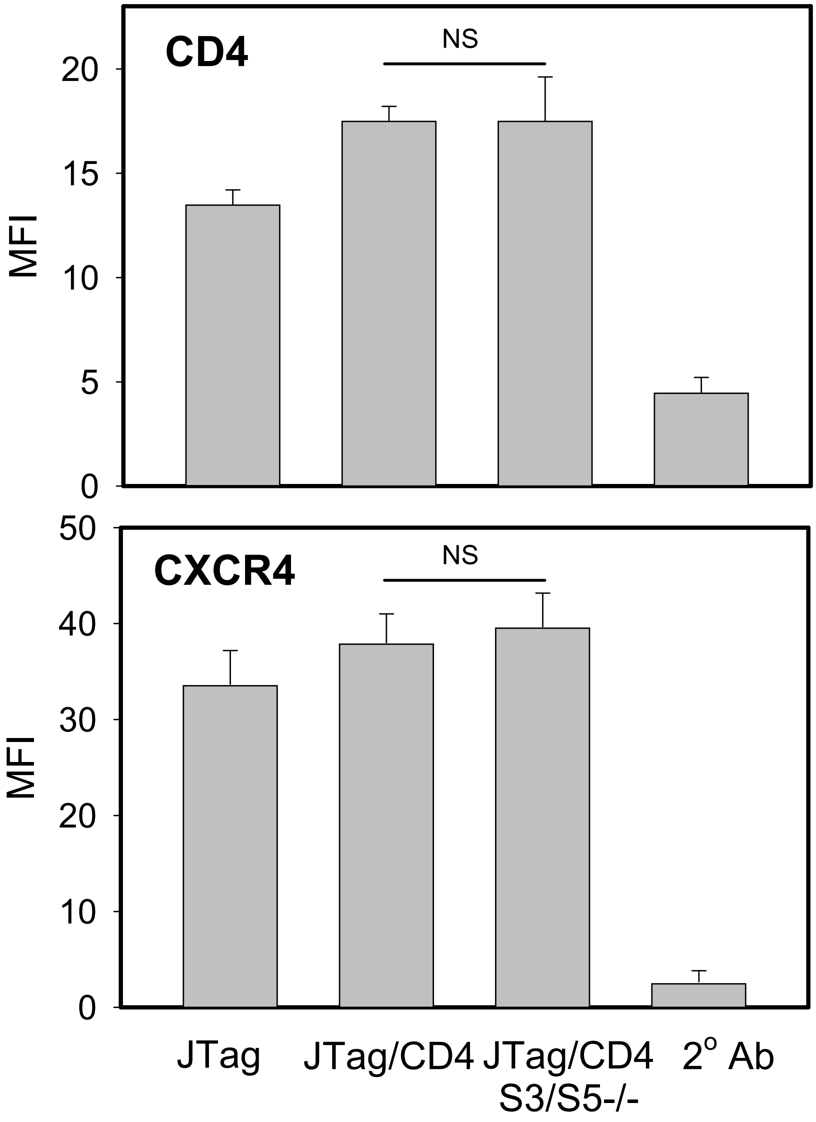

Suppl. Fig. S2. Knockout of SER3 and SER5 does not alter CD4 or CXCR4 expression in JTag cells. Expression of CD4 (A) and CXCR4 (B) on parental JTag/CD4 cells and JTag/CD4 cells depleted of SER3 and SER5 (JTag/CD4/S3/S5/-/-) was measured by flow cytometry, using SIM2 and 12G5 antibodies, respectively. Control samples were incubated with secondary antibodies only (2° Ab). Mean fluorescence intensity (MFI) and SD from 2-3 independent experiments are plotted. Statistical analysis was performed using Student's t-test.

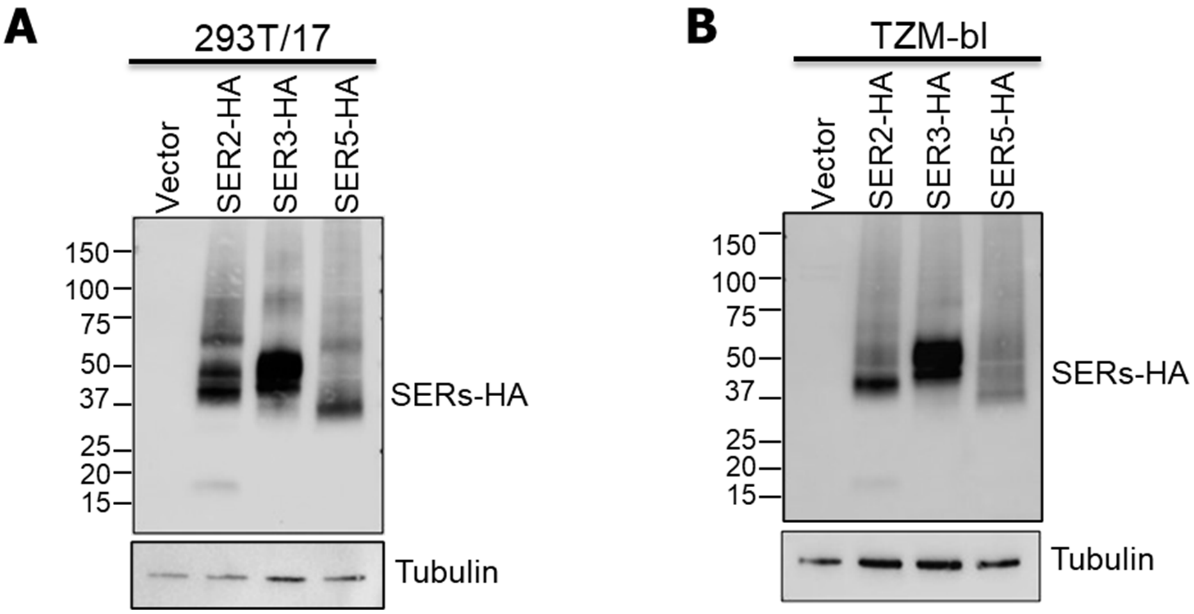

Suppl. Fig. S3. SER2, SER3 and SER5 expression levels in transfected cells. Western blot analysis of the cellular levels of SERs-HA in 293T/17 (A) and TZM-bl (B) cells transiently transfected with the pBJ5 vector expressing SER2, SER3 or SER5 tagged with HA. Cell lysates were obtained and processed 36 h post-transfection, as described in Material and Methods. Tubulin (loading control) blots are shown below.

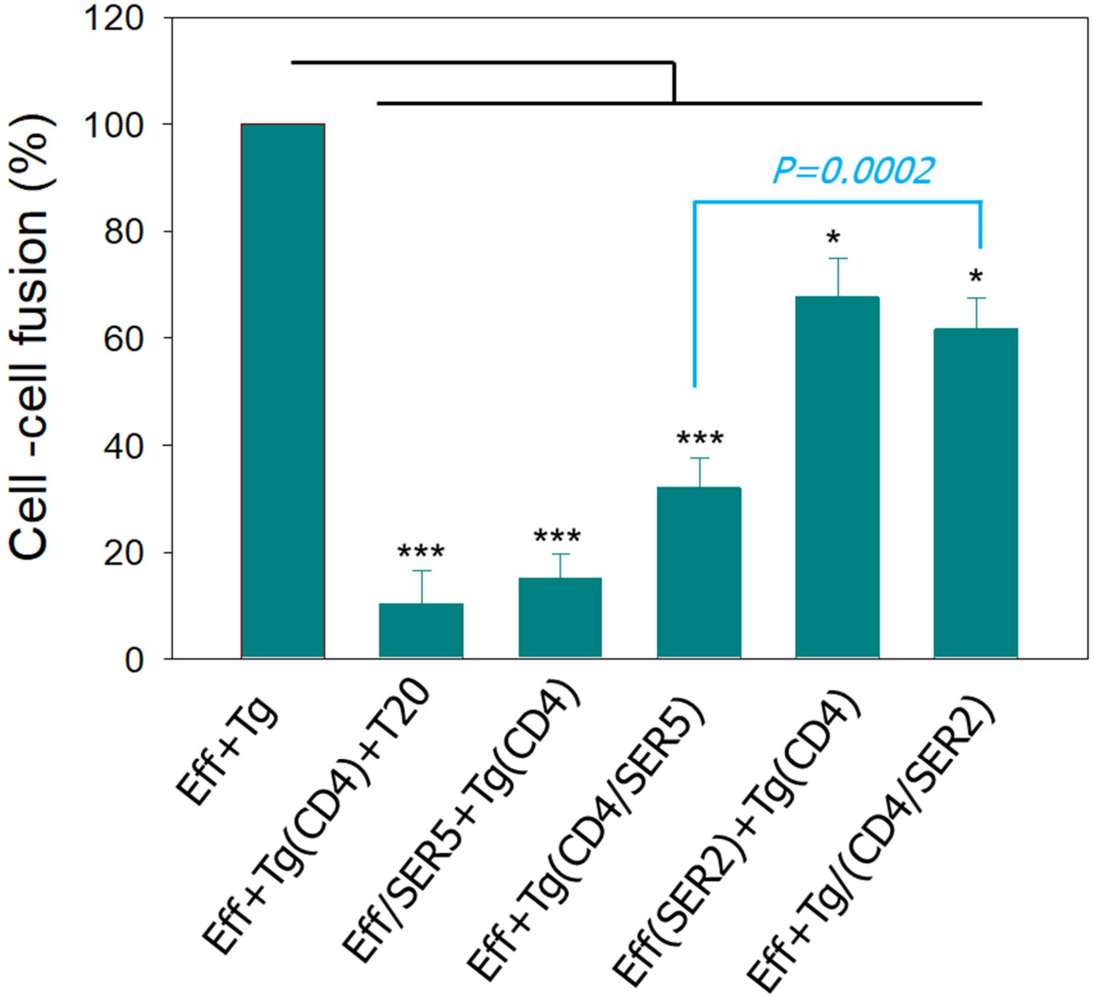

Suppl. Fig. S4. Inhibition of HIV-1 Env-mediated cell fusion by SER5 measured using a split-luciferase assay. Effector (Eff) HEK293 cells stably expressing the DSP8-11 fragment were transfected with HIV-1 HXB2 Env, while target (Tg) HEK293 cells stably expressing the DSP1-7 fragment were transfected with CD4, with or without co-transfection with SER5 or SER2. In control experiments, cell fusion was blocked with the inhibitory peptide T20 (40 nM). Effector and target cells were pre-mixed, allowed to adhere to slides, loaded with the Enduren substrate, and incubated for 3 hours at 37 °C to allow fusion. The extent of cell fusion was measured by a resulting Renilla luciferase signal (see Materials and Methods) using a plate reader. Data are means ± SEM from four independent experiments, each performed in triplicate. Statistical analysis was performed using Student's t-test.

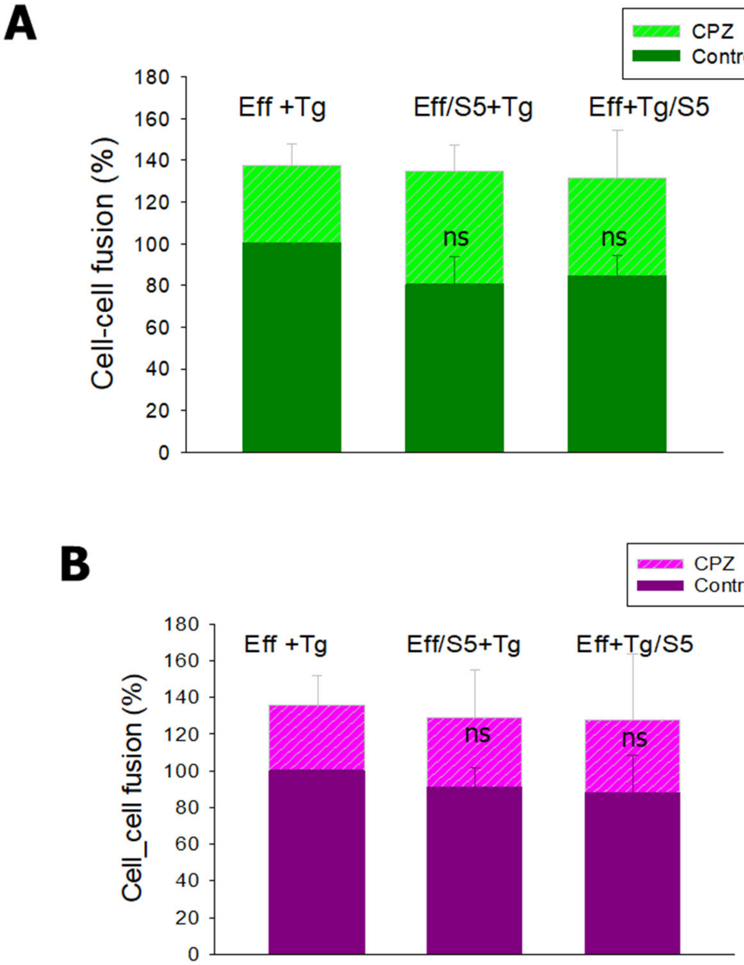

Suppl. Fig. S5. Effect of SER5 on the PR8/34 Influenza HA-mediated cell-cell fusion. Effector (Eff) COS7 cells (A) or HEK293T cells (B) transfected with the Influenza hemagglutinin (HA, PR8/34 strain), with or without SER5, were pretreated with trypsin (0.01 mg/mL) and neuraminidase (0.1 mg/mL) for 5 minutes at room temperature to cleave/activate HA. Cells were washed and co-incubated for 30 min at room temperature with target (Tg) TZM-bl cells transfected or not with a SER5 vector. Fusion was initiated by a 10-minute exposure to pH 4.8 buffer at room temperature, followed by incubation at neutral pH for 1 hour at 37 °C. To rescue cell fusion trapped at a hemifusion stage, cells were exposed to 0.5 mM chlorpromazine (CPZ) for 1 min at room temperature. The extent of cell-cell fusion was measured by a microscopy assay (see Materials and Methods) and normalized to that between control Eff and Tg cells. Plotted are means and S.D. from two independent experiments performed in triplicates. Statistical analysis was performed using Student’s t-test.

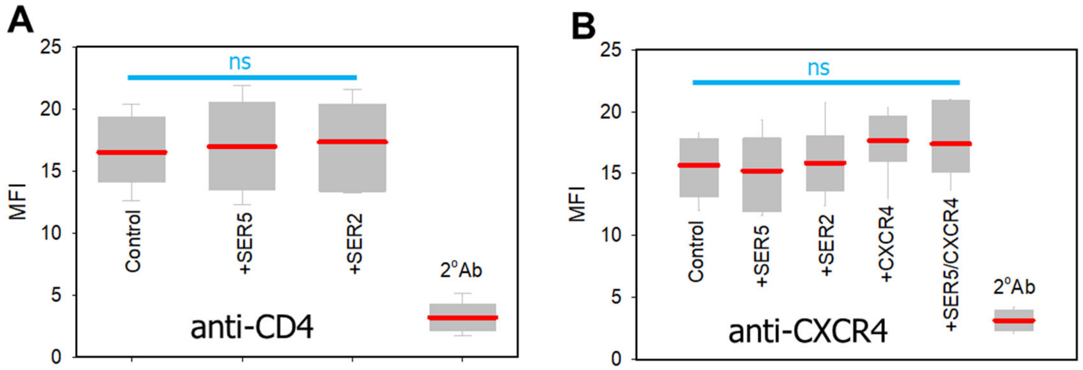

Suppl. Fig. S6. SER5 does not affect cell surface expressions of CD4 and CXCR4. Expression of CD4 (A) and CXCR4 (B) on TZM-bl cells transfected or not with SER5 or SER2 was measured by flow cytometry, using SIM2 and 12G5 antibodies, respectively. (B) Where indicated, TZM-bl cells were transfected with CXCR4 or co-transfected with CXCR4 and SER5 plasmids. Control samples were incubated with secondary antibodies only (2º Ab). Mean fluorescence intensity (MFI) and SEM from four independent experiments, along with quartiles (gray boxes) are plotted. Statistical analysis was performed using Student's t-test.

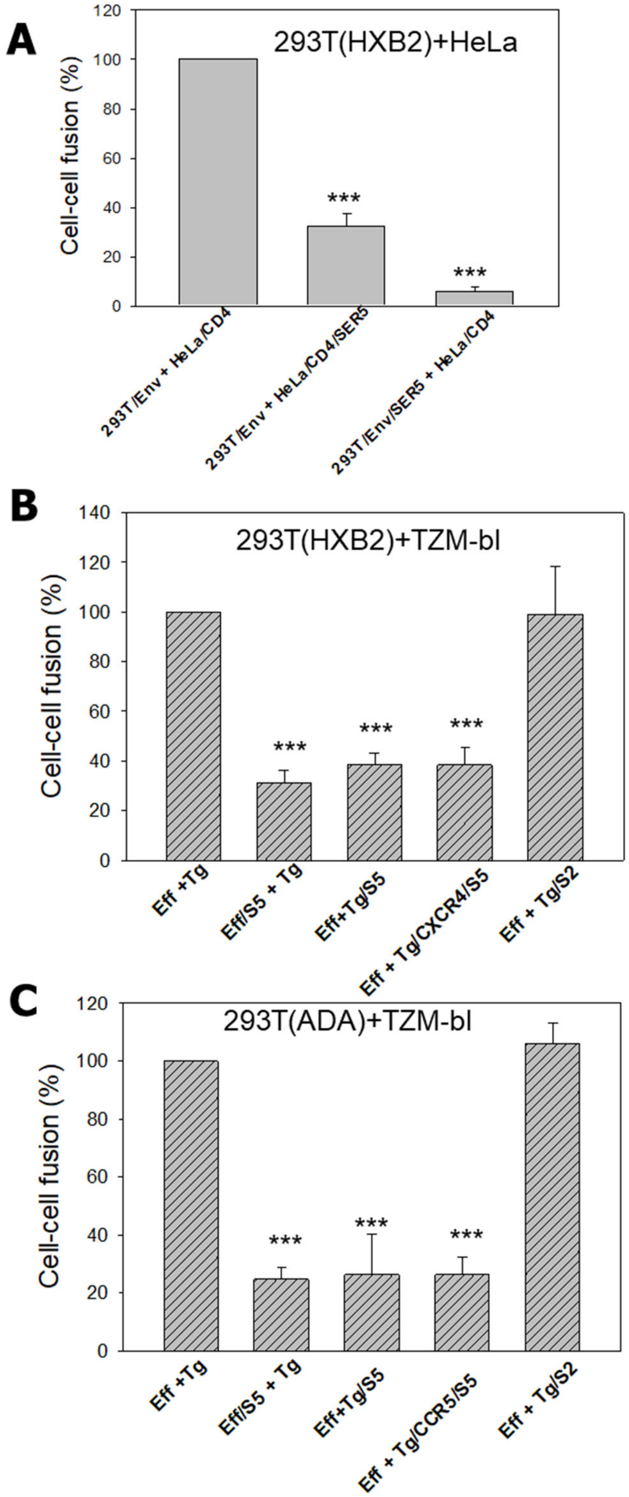

Suppl. Fig. S7. CD4 or coreceptor overexpression does not rescue HIV-1 Env-mediated fusion with SER5-expressing cells. Effector HEK293T cells were transfected with either HIV-1

HXB2 (A, B) or ADA (C) Env glycoprotein-expressing vectors and co-transfected or not with a SER5 plasmid. (A) Target HeLa cells were transfected with CD4, with or without SER5 expressing plasmid, and fused with HEK293T cells expressing HXB2 Env. (B) 293T cells expressing HXB2 Env (and SER5, where indicated) were fused with TZM-bl cells expressing or not SER5 or SER2. Where indicated, TZM-bl cells were co-transfected with a CXCR4 vector. (C) HEK293T cells expressing ADA Env (and SER5, where indicated) were fused with TZM-bl cells expressing or not SER5 or SER2. Where indicated, TZM-bl cells were co-transfected with CCR5. Effector and target cells were pre-mixed and incubated for 3 hours at 37 °C to allow fusion. Fusion activity was quantified by a microscopy-based assay. Data are means ± SEM from three independent experiments, each performed in duplicate. Statistical analysis was performed using Student's t-test.

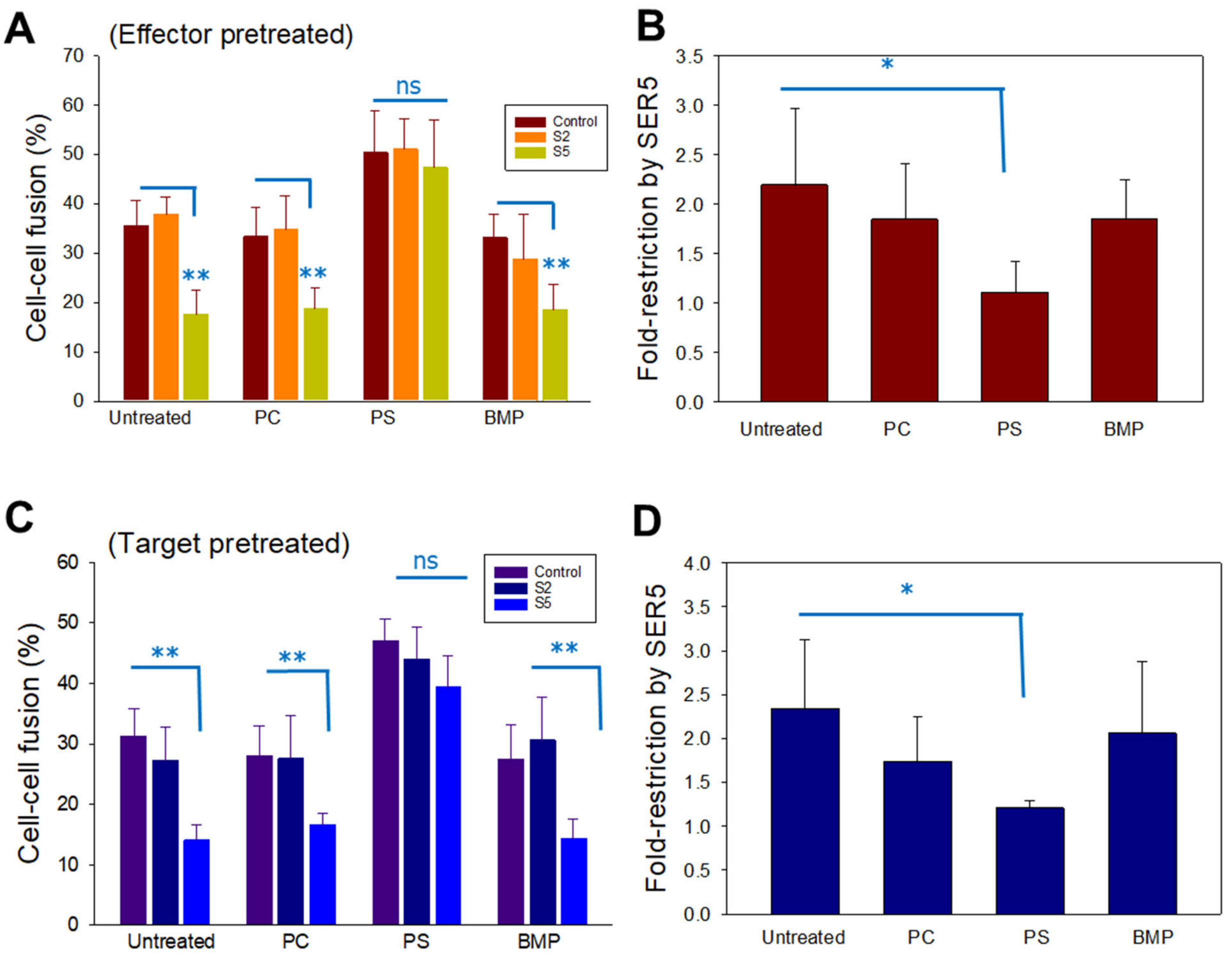

Suppl. Fig. S8. **Exogenous lipids can rescue HIV-1 Env-mediated fusion with SER5-expressing cells.** TF228.1.16 cells stably expressing BH10 Env were fused with TZM-bl transfected or not with SER2 or SER5. Effector cells (A, B) or target cells (C, D) were pretreated separately for 20 minutes at room temperature with PBS supplemented with BSA (1 mg/mL, "Untreated") and with freshly suspended lipids, DOPC, DOPS, DOPG, or BMP (10 µg/mL). Cells were washed twice with PBS/BSA, mixed with respective target or effector cells, and co-incubated for 2.5 hours at 37 °C to allow fusion, which was quantified by fluorescence microscopy. The impact of exogenous lipids on SER5-mediated restriction of fusion is plotted as fold-restriction in panels C and D. Data are means ± SEM from four independent experiments, each performed in duplicate. Statistical analysis was performed using Student's t-test.

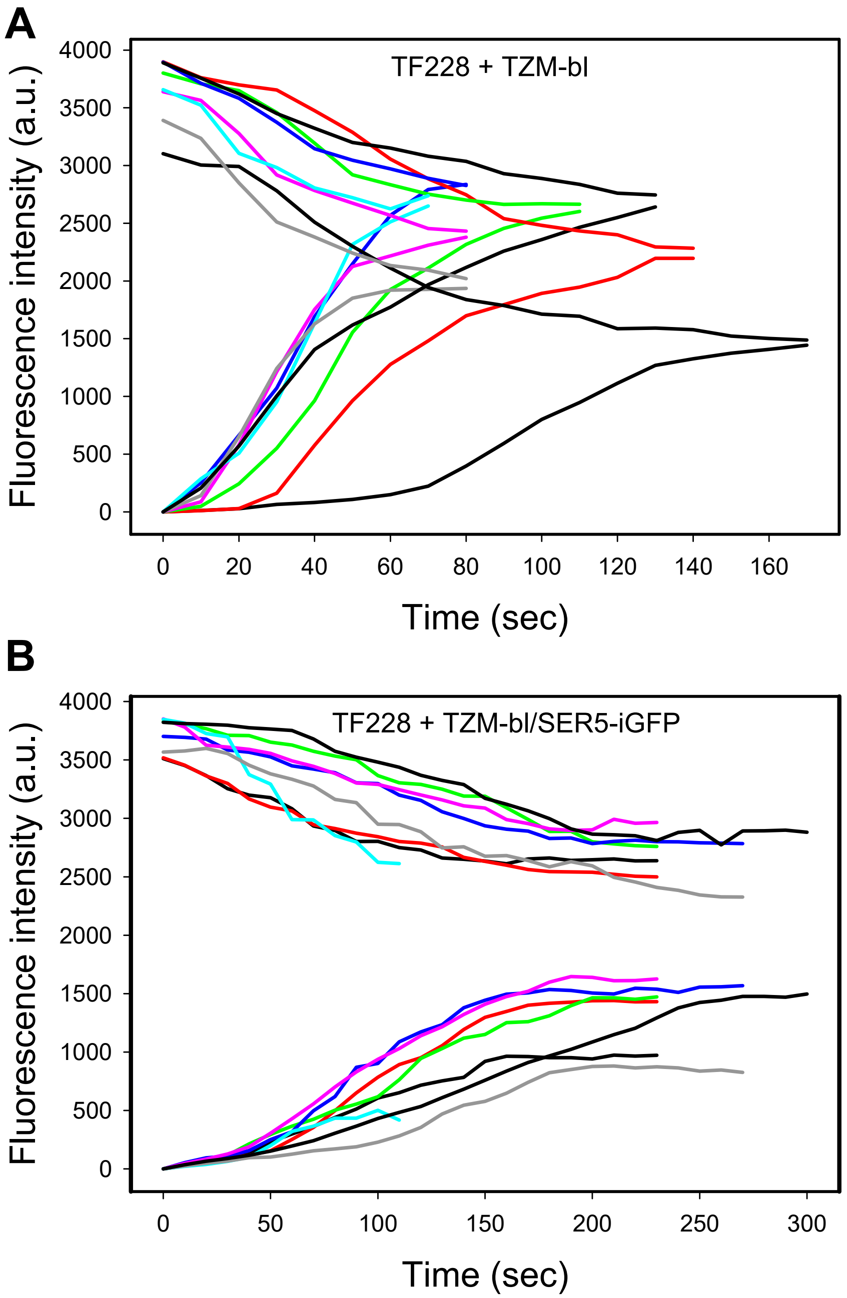

Suppl. Fig. S9. Examples of calcein redistribution between effector and target cells. Effector TF228.1.16 cells were loaded with calcein and incubated with target cells TZM-bl cells mock-transfected (A) or transfected with SER5-GFP (B) for 2.5 h at 23 ºC to establish a temperature arrested stage. Fusion was triggered by quickly shifting to 37 ºC and monitored as calcein redistribution from effector to target cells. Descending traces show reduction in integrated calcein signal intensity of effector cells, while ascending traces show the appearance of calcein in target cells (using the same color for a given effector/target cell pair).

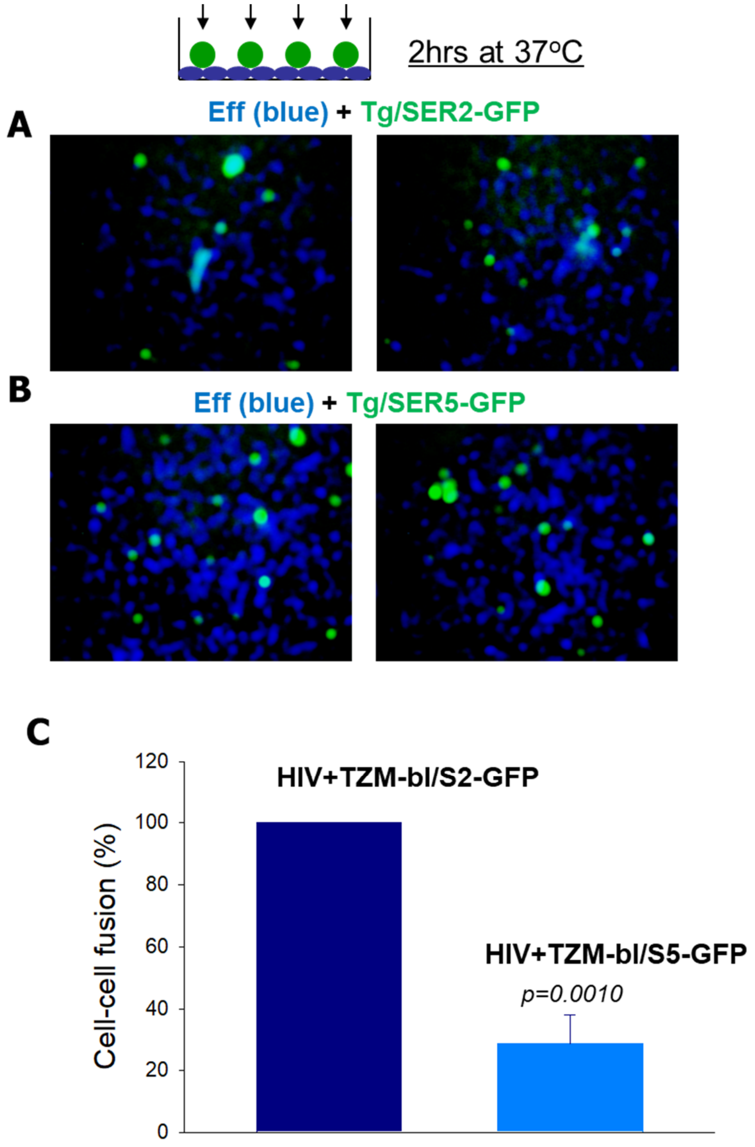

Suppl. Fig. S10. **Validation of GFP-tagged SER5 and SER2 constructs.** HEK293T effector cells, transfected with HIV-1 HXB2 Env, were plated to reach full confluency. On the day of experiment, effector cells were labeled with CMAC (blue). Target TZM-bl cells were transfected with either SER2-GFP (A) or SER5-GFP (B). Target TZM-bl cells (green) were detached using a non-enzymatic solution, suspended, overlaid onto the effector cell monolayer, and incubated for 2 hours at 37 °C to allow fusion. After incubation, unbound target cells were gently washed off with PBS, and fused cells were identified by co-localization of GFP and CMAC signals. (C) Fusion was quantified as the percentage of fused cells relative to total GFP-positive target cells. Results from three independent experiments are shown. Statistical analysis was performed using Student's t-test.

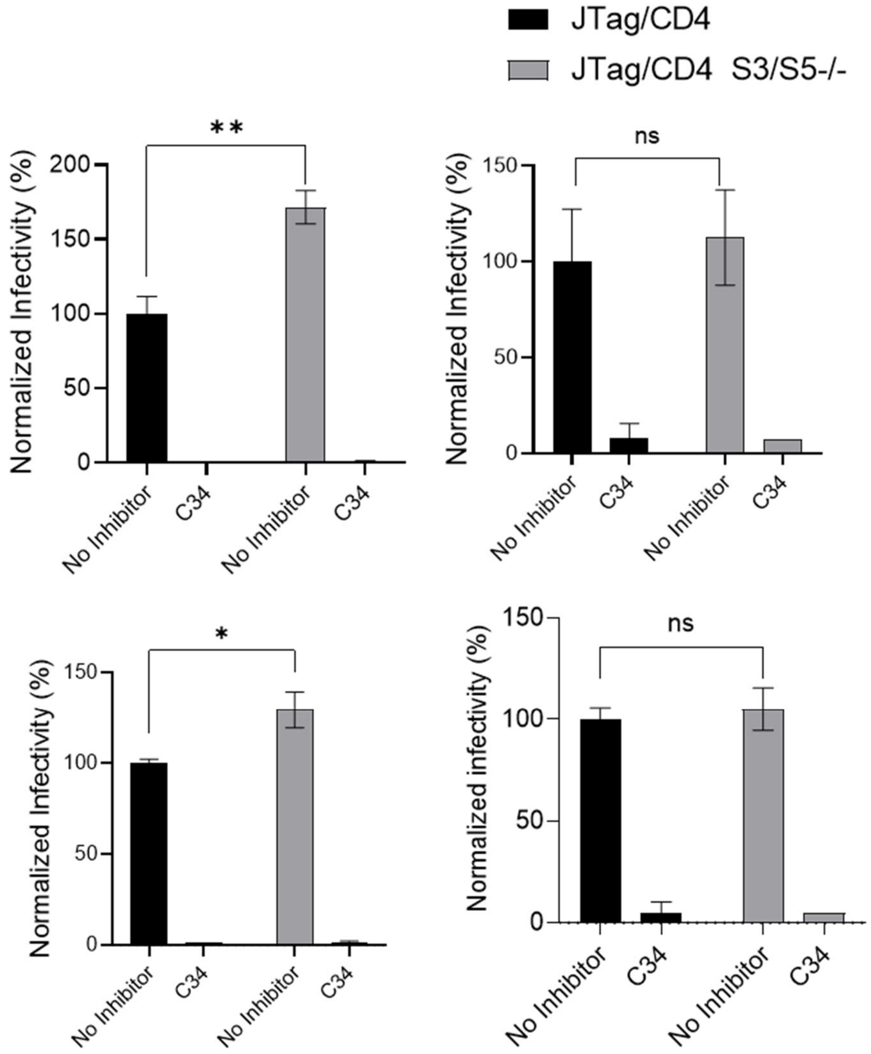

Suppl. Fig. S11. HXB2 pseudovirus infection of JTag/CD4 and JTag/CD4 S3/S5-/- cells. HXB2/NL4-3.Luc.E-R- pseudoviruses were used to inoculate $1 \cdot 10^5$ cells (2 IU/cell) by centrifugation at 4 °C for 30 min at 1550×g. Cells were washed and incubated at 37 °C, 5% CO2 for 48 h to allow the infection. The plots show mean and S.D. for each of 4 independent experiments, each performed in triplicate. Control samples used 1 μM C34 peptide. Statistical analysis was performed using Student's *t*-test. *, $p < 0.05$; **, $p < 0.01$; ns, not significant.
